# Case report of tracheobronchial injuries after acid ingestion: CT findings with serial follow-up: Airway complication after acid ingestion

**DOI:** 10.1097/MD.0000000000023586

**Published:** 2020-12-11

**Authors:** Nokjung Kim, Han Na Lee, Jung Im Kim, So Youn Shin, Sung Wook Kang

**Affiliations:** a Department of Radiology, Kyung Hee University Hospital; b Department of Radiology, Kyung Hee University Hospital at Gangdong, College of Medicine, Kyung Hee University; c Division of Pulmonary and Critical Care Medicine, Department of Internal Medicine, Kyung Hee University Hospital at Gangdong, Seoul, Republic of Korea.

**Keywords:** acid aspiration, chest CT, tracheobronchial injury

## Abstract

**Rationale:**

Tracheobronchial injury from acid ingestion is a less reported clinical presentation than injury of the gastrointestinal tract, but it can occur due to direct exposure from acid aspiration and cause fatal complications.

**Patient concerns:**

A 43-year-old man presented to the emergency department after ingesting nitric acid complaining of chest pain and dyspnea.

**Diagnoses:**

The initial chest computed tomography (CT) images revealed an acute lung injury related to acid aspiration. The follow-up chest CT showed acute and late tracheobronchial injures.

**Interventions:**

Bronchoscopy showed deep caustic airway injuries consisting of hemorrhage, sloughing of the mucosa, and ulceration of the trachea and left-side bronchial tree.

**Outcomes:**

Progressive narrowing of the left main bronchus with total collapse of the left lung occurred as a late complication of acid ingestion.

**Lessons:**

Tracheobronchial injury should be considered in cases of aspiration pneumonia after acid ingestion; chest CT can be used to detect and assess acute and late complications of tracheobronchial injuries.

## 1 Introduction

Caustic ingestion in adults is a rare, life-threatening condition that causes injuries from the laryngopharynx to the upper gastrointestinal tract. The severity of tissue damage after caustic ingestion depends on the agents nature (acid vs alkali), volume, concentration, and contact time.^[1]^ Tracheobronchial injury after caustic ingestion is uncommon, but can result in severe complications, including airway necrosis, perforation, or progressive luminal narrowing.^[2,3]^ Herein, we report a rare case of tracheobronchial injury after acid ingestion and the accompanying CT findings.

## 2 Case description

A 43-year-old male presented to our emergency department approximately 5 hours post ingestion of approximately 300 ml of nitric acid with suicidal intentions. The patient complained of chest pain, dyspnea, and a severe sore throat. On physical examination, erosions, and erythema were found in the oropharynx.

**Figure 1:**
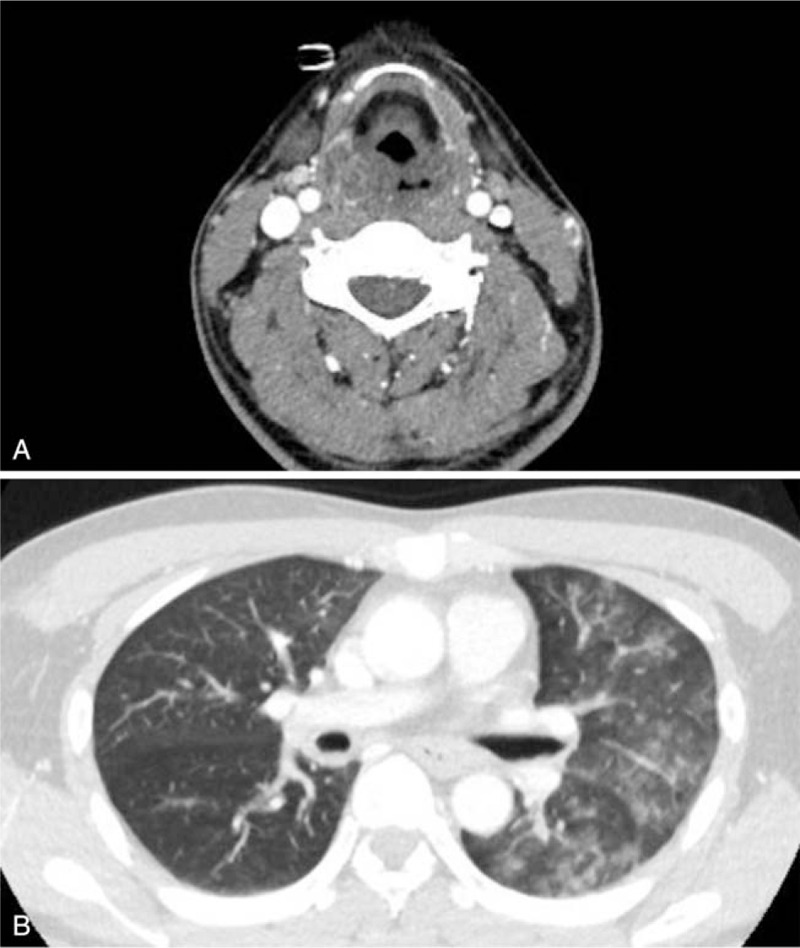
Chest CT findings after acid aspiration on the day of exposure. A. Swelling of hypopharynx and epiglottis is shown. B. Multifocal peribronchial ground-glass opacities shown in the left lung suggest acute lung injury related to acid aspiration.

The initial chest CT revealed diffuse swelling of the upper airway and gastrointestinal tract involving the epiglottis, oropharynx, and esophagus. Multifocal, patchy, ground-glass opacities were also noted, primarily in the dependent areas of the right upper lobe and left lung, indicating possibly related to acid aspiration (Fig. 1). Intubation with mechanical ventilation was subsequently performed in the intensive care unit due to respiratory distress.

**Figure 2:**
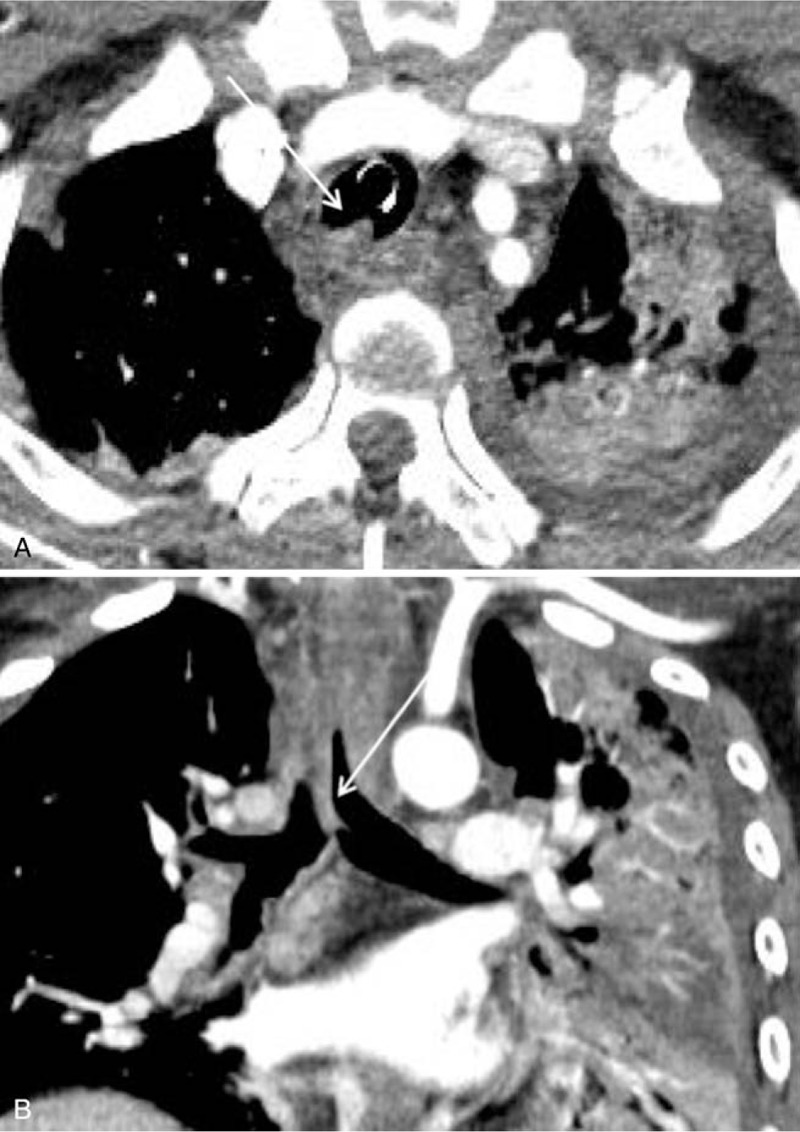
Chest CT findings of acute airway injury 5 days post ingestion. A, B. Interval-developed endobronchial lesions from the trachea to the left main bronchus with tenting shape suggest mucosal injury with swelling.

**Figure 3:**
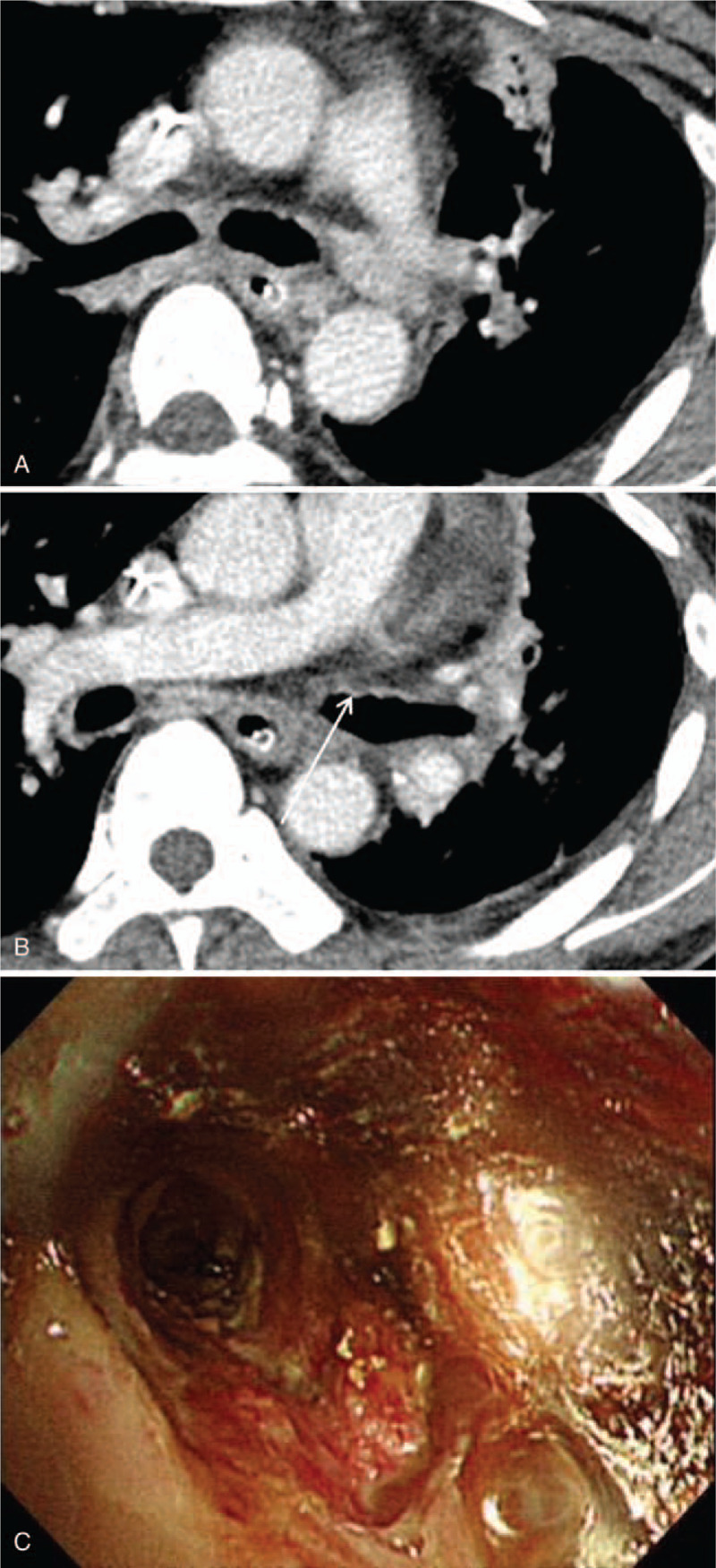
Chest CT findings of airway injury 1 month post acid ingestion. A, B. Irregular surface with wall thickening of the left main bronchus is shown on chest CT C. Multiple white patches and erosions are shown on the left main bronchus.

A follow-up chest CT 5 days post ingestion showed development of continuous endobronchial lesions with a tenting shape on the posterior wall of distal tracheal to left main bronchus and these lesions were correlated with mucosal nodularities on the bronchoscopy (Fig. 2A, B). One month later, the chest CT showed an irregular surface on the left main and left upper lobar bronchi (Fig. 3A, B), while preserving the patency of bronchial lumen. Further evaluation with bronchoscopy on the same day showed deep caustic airway injuries manifesting as hemorrhage, sloughing of the mucosa, and ulceration along the trachea and left bronchial tree (Fig. 3C). These changes were more prominent on the left side of the airway, the same side with prominent lung opacities, noted previously.

**Figure 4:**
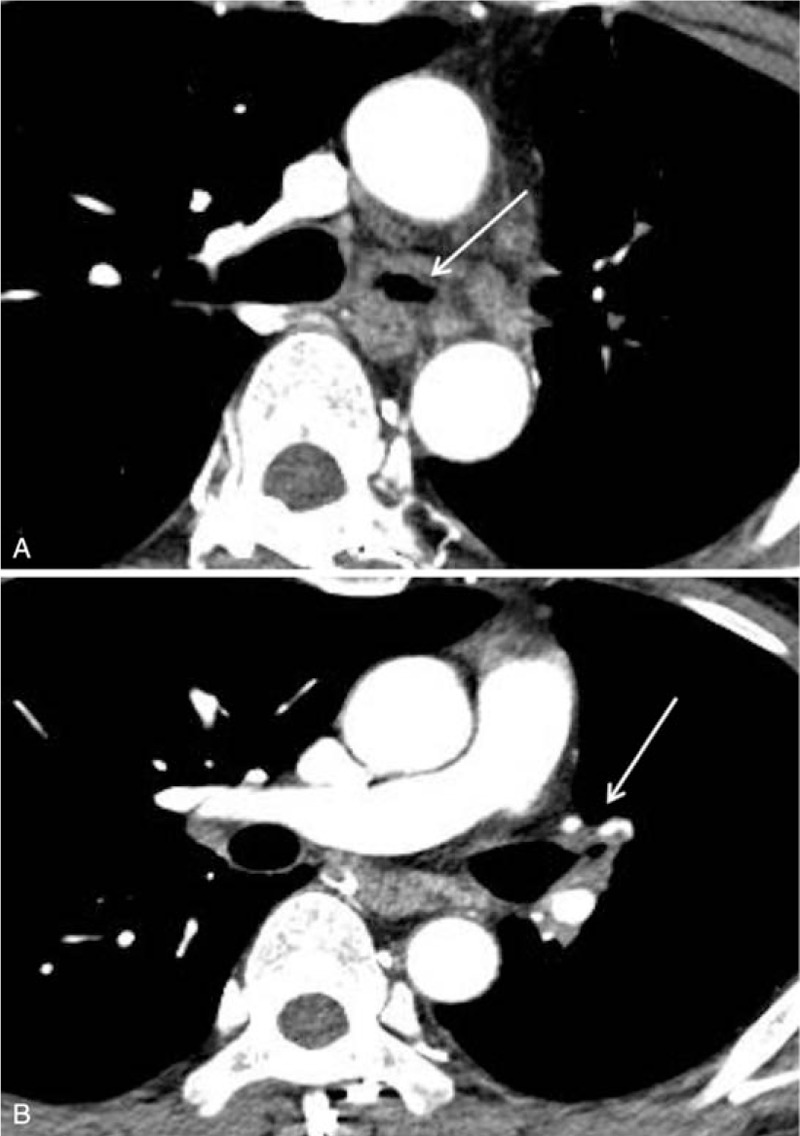
Chest CT findings of late airway complication 4 months post acid ingestion. A, B Luminal narrowing of the left main and left upper lobar bronchi with bronchial wall thickening is seen on chest CT (arrows).

**Figure 5:**
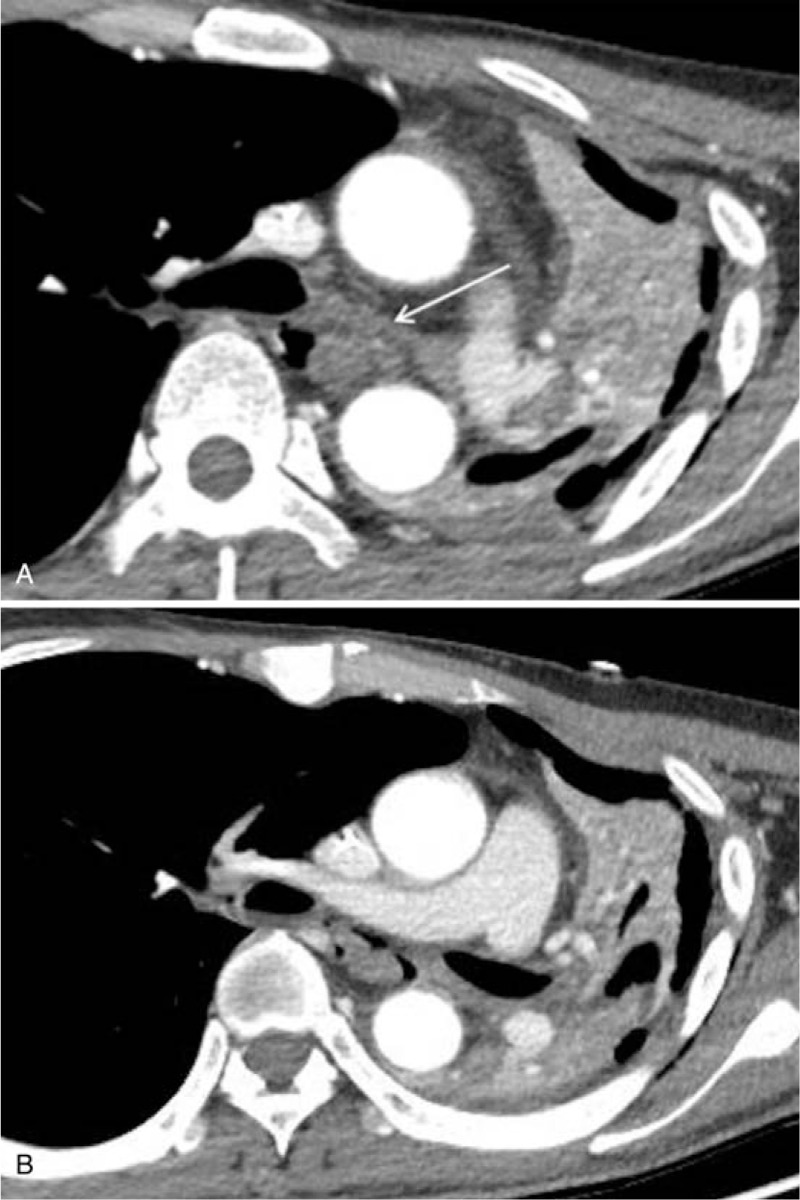
Chest CT findings of late airway complication 6 months post acid ingestion. A, B. Progressive narrowing of the left main bronchus (arrow) is shown on chest CT; resultant total collapse of the left lung is also seen.

An additional chest CT was performed 4 months post ingestion that showed severe tracheobronchial wall thickening and luminal narrowing in the left main and left upper lobar bronchi with irregular stenosis and post-stenotic dilatation of the airway (Fig. 4A, B). A follow-up chest CT 6 months post ingestion showed progression of the luminal narrowing of the left main bronchus with resultant total collapse of the left lung (Fig. 5A, B). The patient, reportedly, could perform limited daily activities with a tracheostomy tube in place to prevent aspiration due to impairment of laryngeal function.

This study was reviewed and approved by our hospitals institutional review board (2020-06-012) and informed consent was waived.

## 3 Discussion

We have reported a rare case of tracheobronchial injuries from acid aspiration with serial CT findings. The patient lost near total left lung function due to severe stenosis of the left main bronchus, a late complication from the acid aspiration. To our knowledge, this is the first report of tracheobronchial injuries from nitric acid aspiration.

Corrosive tracheobronchial injury is a less common clinical entity than corrosive injury of the GI tract, but it can result in chemical tracheobronchitis and, importantly, fatal complications such as tracheobronchial necrosis, perforation, or stenosis.^[2,4]^ Corrosive airway injuries can be caused by aspiration or by necrosis extension from the neighboring esophagus.^[1]^ Corrosive airway injury by aspiration occurs more frequently with acids than alkaline ingestions, since it may be related to the bad taste of acid materials and resultant gagging, choking, and vomiting of the ingested materials.^[5]^

The acute phase of corrosive tracheobronchial injury includes airway hemorrhage and obstruction due to tissue sloughing, exudation, ulceration, and granulation tissue growth, potentially due to direct exposure of the airway to concentrated liquid acid.^[3,4]^ Later complications include upper airway fibrosis with progressive luminal narrowing and mucociliary dysfunction.^[3]^ The development of large airway stenosis, as in our case, can cause wheezing or eventual loss of ipsilateral lung function.

Chest CT tends to underestimate the severity of acute phase of corrosive airway injury,^[6,7]^ and we also did not detect acute subtle changes of tracheobronchial tree on chest CT. When retrospectively reviewing chest CT images, we found continuous endobronchial lesions and luminal irregular surfaces on the distal trachea to left main bronchus; these findings correlated with the bronchoscopic findings of mucosal swelling, nodularities, ulceration, and necrotic tissue debris. In particular, if aspiration pneumonia was observed on the initial chest CT after acid ingestion, it is necessary to mention even subtle airway changes in acute stage to recognize and reduce potential late complication of airway. Late airway complication detected on chest CT include airway luminal stenosis due to ongoing process of healing and fibrosis^[3,8]^ Chest CT can evaluate extent and degree of airway stricture that is difficult to approach for bronchoscopy.

The mortality rates of corrosive injuries are generally reported to be 10% to 20%,^[9]^ but it can be as high as 45% or 75% when airway injury of tracheal bronchial necrosis occurs.^[4,10]^ Tseng et al reported that occurrence of aspiration pneumonia suggesting airway injury increased the mortality rate in acid injured patients, and 2 of 6 survivors of aspiration pneumonia after acid ingestion later developed laryngeal and tracheal sequelae.^[11]^

In conclusion, careful evaluation for possible airway injury after acid ingestion is required; particularly if an initial chest CT suggests aspiration pneumonia after acid ingestion. Chest CT can assist in detecting and evaluating corrosive airway injures and the resulting complications.

## Author contributions

**Conceptualization:** Han Na Lee.

**Data curation:** Nokjung Kim.

**Methodology:** Jung Im Kim, So Youn Shin.

**Resources:** So Youn Shin.

**Validation:** Jung Im Kim.

**Writing – original draft:** Nokjung Kim, Han Na Lee.

**Writing – review & editing:** Han Na Lee, So Youn Shin.
